# The efficacy of the cyclin-dependent kinase 4/6 inhibitor in endometrial cancer

**DOI:** 10.1371/journal.pone.0177019

**Published:** 2017-05-04

**Authors:** Tomohito Tanaka, Yoshito Terai, Keisuke Ashihara, Satoe Fujiwara, Yoshimichi Tanaka, Hiroshi Sasaki, Satoshi Tsunetoh, Masahide Ohmichi

**Affiliations:** Department of Obstetrics and Gynecology, Osaka Medical College, Takatsuki, Japan; University of Navarra, SPAIN

## Abstract

**Background:**

PD-0332991, the selective cyclin-dependent kinase 4/6 inhibitor palbociclib, causes cell cycle arrest by inhibiting phosphorylation of retinoblastoma (Rb) protein. The aim of this study was to evaluate the therapeutic potential of PD-0332991 in endometrial cancer.

**Methods and findings:**

Four human endometrial cancer cell lines, ECC, HEC1A, HEC108 and TEN, were treated with PD-0332991 and their function was evaluated. *In vivo*, the therapeutic efficacy was evaluated in a model of subcutaneous endometrial cancer. An immunohistochemical analysis was performed in 337 endometrial cancer specimens. A proliferation assay revealed that 2 of the 4 cell lines that expressed Rb were sensitive to PD-0332991 with an IC50 of 0.65 μM (HEC1A) and 0.58 μM (HEC108), respectively. Both cell lines had G0/G1 cell cycle arrest after treatment with PD-0332991 according to flow cytometry. *In vivo*, PD-0332991 had antitumoral efficacy with a reduction in the activity of Ki67 and phosphorylation of Rb. Immunohistochemical analyses revealed that the positive rate of Rb was 67.7%, however, there was no significant relationship between the expression levels of Rb and the tumor grade.

**Conclusions:**

PD-0332991 had therapeutic potential against endometrial cancer cell lines expressing Rb protein. Our immunohistochemical analysis revealed that approximately 70% of patients with endometrial cancer might have therapeutic indications for PD-0332991. Of note, the tumor grade had no impact on the indications for treatment.

## Introduction

Endometrial cancer is the most common gynecologic malignancy in the United States as well as other developed countries [1]. It has been proposed that endometrial carcinomas are pathogenetically divided into type 1 and type II tumors [2]: estrogen-dependent neoplasms called type I account for 80–85% of all cases and estrogen-independent tumors called type II make up the remaining 10–15% of the cases [3, 4]. Most patients (84%) with endometrial cancer have no evidence of extrauterine spread [5], however, 10–15% of those patients will experience recurrence after surgical treatment [6, 7]. Moreover, 16% of women with endometrial cancer, which is only a small proportion, present with advanced disease [5]. For those patients with recurrent or advanced disease, radiotherapy or chemotherapy may be performed, however, a desirable prognosis has not yet been attained and new breakthrough treatment is expected.

PD-0332991, known as palbociclib, is a selective cyclin-dependent kinase (CDK) 4/6 inhibitor which induces cell cycle arrest with reduced retinoblastoma (Rb) phosphorylation [8]. The compounds CDK 4/6 and cyclin D1 release transcription factor E2F from Rb, which results in phosphorylation of Rb and subsequent cell proliferation [9, 10]. INK4 family members, including p15/INK4B (CDKN2B), p16/INK4A (CDKN2A), p18/INK4C (CDKN2C) and p19/INK4D (CDKN2D), act as inhibitors of CDK 4/6 [11, 12]. Several studies have shown that PD-0332991 has sensitivity to cell lines expressing Rb protein [13–16]. Furthermore, the most sensitive cell lines to PD-0332991 had both p16 loss and cyclin D1 amplification [14, 15]. In breast cancer, the expression of Rb is higher in luminal A subtype tumors and PD-0332991 is more effective in these cell lines [13]. For these reasons, a clinical study for breast cancer was designed considering several factors such as Rb status, hormone receptor status, p16 expression and cyclin D1 amplification [17–21]. However, a phase II trial (PALOMA1/TRIO1) suggested that patient selection based on cyclin D1 amplification and p16 loss was unlikely to further improve the patient outcome over the use of the estrogen-receptor (ER) and HER2 status alone [19]. A subsequent phase III trial (PALOMA2/PALOMA3) was designed for patients with ER+/HER2 advanced breast cancer. PALOMA2 was completed and the results should be available soon. PALOMA3 was stopped early due to efficacy issues [20]. Unfortunately, there have been no reports regarding the effect of PD-0332991 on endometrial cancer; thus the aim of this study was to evaluate the therapeutic potential of PD-0332991 in endometrial cancer.

## Materials and methods

### Patients and tissue samples

The present study included 337 Japanese patients with endometrial cancer who were treated at Osaka Medical College between 2002 and 2010. Patients were eligible for inclusion in the study when they met the following criteria: (1) they had undergone hysterectomy as an initial treatment; and (2) they had sufficient clinical data regarding the oncologic outcome including the date of recurrence. All patients were staged according to the International Federation of Gynecology and Obstetrics (FIGO) criteria. The histological subtype was assigned according to the criteria of the World Health Organization classification. Most patients who had deep half myometrial invasion or high grade tumors received postoperative adjuvant therapy involving chemotherapy or pelvic irradiation. The present study was approved by the institutional review board (IRB) of Osaka Medical College. Written informed consent was obtained from some patients for the use of their tissue samples and clinical records in the present study. The other patients provided their written informed consent at the time of primary surgery to use their tissue samples and clinical records for an IRB-approved study; the IRB approved this consent procedure.

### Cell culture

We used four endometrial cancer cell lines in this study. ECC, HEC1A and HEC108 cells were obtained from the American Type Culture Collection (ATCC, Rockville, MD, USA), and TEN cells were provided by the RIKEN BRC through the National Bio-Resource Project of the MEXT, Japan (Ibaraki, Japan). All cell lines were grown in dishes (Becton Drive, Franklin Lakes, NJ, USA) in DMEM supplemented with 10% charcoal stripped fetal bovine serum (FBS) (Equitech-Bio, Kerrville, TX, USA) (growth medium) in an atmosphere of 5% CO_2_ at 37°C. Serum-free DMEM was used for cell starvation. ECC, HEC1A and HEC108 cells were derived from well-differentiated, grade 2 and grade 3 endometrioid carcinomas of the uterus, respectively. TEN cells were derived from an endometrial clear cell carcinoma. ATCC and RIKEN BRC routinely authenticate their cell lines using a short tandem repeat (STR) polymorphism profiling analysis. All experiments were performed at passages < 15–20.

### Western blot analysis

After serum starvation for 12 hours, cells were treated with increasing concentrations and exposure times of PD-0332991 (Sigma-Aldrich, St. Louis, MO, USA). Cells with or without treatment were washed twice with ice-cold phosphate-buffered saline, lysed, and separated into cytoplasmic and nuclear fractions using the Nuclear Extract Kit according to the manufacturer’s instructions (Active Motif, Carlsbad, CA, USA). To detect Rb, phospho-Rb, p15, p16, p18, p19 and cyclin D1 proteins, we separated the fractions using SDS polyacrylamide gel electrophoresis and electrotransferred the proteins to nitrocellulose membranes. Western blot analyses were performed with various specific primary antibodies including Rb (4H1, 9309, Cell Signaling Technology, Danvers, MA), phospho-Rb (Ser780, 8180, Cell Signaling), p15 (p15 INK4b, ab53034, Abcam, Cambridge, MA, USA), p16 (CDKN2A/P16INK4a, ab54210, Abcam), p18 (p18INK4c, ab3216, Abcam), p19 (p19 INK4d, ab102842, Abcam) and β-actin (4970, Cell Signaling). The immunoreactive bands in the immunoblots were visualized with horseradish peroxidase-coupled goat anti-rabbit or mouse immunoglobulin using an enhanced chemiluminescence Western blotting system (ECL Plus, GE Healthcare Life Sciences, Pittsburgh, PA, USA). Nonspecific antigen sites were blocked with 10% bovine serum albumin in 1X Tris-buffered saline.

### Proliferation assays

The cells were placed onto 24-well tissue culture plates at a density of 1 × 10^4^ and grown without or with increasing concentrations of PD-0332991 (ranging between 0.001 to 1 μM). Cells were harvested by trypsinization on day 3 and counted using a hemocytometer with trypan blue stain. All experiments were performed three times in triplicate for each cell line.

### Cell cycle analysis

To analyze the cell cycle distribution, cells were plated onto 6-well plates at a density of 2 × 10^5^ cells per well, then cells were cultured in growth media until they reached 60% confluence. The cells were harvested after being incubated for 72 hours in the absence or presence of 1 or 10 μM of PD-0332991, then cell proliferation was evaluated by measuring the distribution of the cells in different phases of the cell cycle by flow cytometry using the Cycle TEST PLUS DNA Reagent kit (BD Pharmingen, San Diego, CA, USA), which is based on the measurement on the DNA content of nuclei labeled with propidium iodide, according to the manufacturer’s instructions. Briefly, cells were trypsinized (250 μL of trypsin buffer) for 10 min at room temperature, and then a trypsin inhibitor (200 μL) and RNase buffer were added and allowed to react for 10 min at room temperature. Finally, propidium iodide staining solution (200 μL) was added and the cells were incubated for 10 min in the dark on ice. Samples were immediately analyzed on the EC800 Flow Cytometry Analyzer (Sony Biotechnology Inc., Champaign, IL, USA). The Flowjo version 9 software program (Tree Star, Inc., Ashland, OR, USA) was used for the cell cycle analysis. The experiment was carried out three times, and the ratio of cell cycle distribution was expressed as the mean ± S.D.

### Immunohistochemistry

The specimens were fixed in 10% formalin and embedded in paraffin. Serial sections cut from paraffin-embedded blocks were used for routine histopathology. A 4 μm section was cut from a tissue microarray block and immunohistochemically analyzed for the expression of Rb, phospho-Rb, P16, cyclin D1 and Ki-67. Deparaffinized and rehydrated sections (4 μm) were autoclaved in 0.01 M citrate buffer (pH 6.0) for 15 min at 121°C for antigen retrieval. Endogenous peroxidase activity was blocked with 0.3% solution hydrogen peroxide in methanol for 30 min. Tumor sections were incubated at 4°C for 12 h with an anti-Rb antibody (4H1; 1:100 dilution, Cell Signaling), anti-phospho-Rb antibody (Ser807/811; 1:200 dilution, Cell Signaling), anti-CDKN2A/p16INK4a antibody (ab54210; 1:1000 dilution, Abcam), anti-Cyclin D1 antibody (ab16663; 1:100 dilution, Abcam) and anti-Ki-67 antibody (AB9260; 1:300 dilution, Chemicon, Temecula, CA, USA). The sections were washed with 1X phosphate-buffered saline (PBS) and incubated with Histofine simple stain MAX PO (multi; Nichirei) for 30 min at room temperature. Finally, the sections were washed with 1X PBS and then visualized by incubation with H_2_O_2_/diaminobenzidine substrate solution for 5 min. The sections were counterstained with hematoxylin prior to dehydration and mounting. The evaluation of the immunohistochemical data was performed by two independent pathologists who were blinded to the clinicopathological data. Immunostaining intensity was recorded in three categories: + (weak), ++ (moderate), or +++ (strong). Tumors were considered to be positive for each antibody if more than 10% of the tumor was stained. To determine the labeling index (LI) of Ki-67, Rb and phospho-Rb, the percentage of cells with positive nuclear staining in at least 1200 tumor cells in 3 randomly selected high-power fields, which we selected as typical and well-stained areas, was expressed as the labeling index [22, 23].

### In vivo experiments

Female 6-week-old athymic nude mice (BALB/c Slc-nu/nu) were used for the tumor experiments. The mice had access to sterile food pellets and water *ad libitum* on a 12-hour light/dark cycle. All procedures were performed after obtaining approval from the Osaka Medical College Animal Committee, and the institutional guidelines for animal welfare and experimental conduct were followed. Briefly, the athymic nude mice (18–22 g) were randomized. The mice were anesthetized with isoflurane, and endometrial cancer cell line fragments (HEC1A and HEC108) were injected subcutaneously in the region of the right axilla using a 26-gauge needle. Treatment was initiated when the tumors reached at least 100 mg in weight. Several doses (vehicle, 50 or 150 mg/kg/day) of PD-0332991 (Aooq BIOSCIENCE, Irvine, CA, USA) were given daily per os for 21 days by gavage as solution in lactate buffer (50 mmol/l) at pH 4.0 based on the mean group body weight [24, 25]. In all experiments, there were 5 mice in the control and each treated group. Tumor volume was monitored over time. After 21 days, the treatment was discontinued and all of the mice were sacrificed by overdose of isoflurane. The tumor were then isolated and the immunohistochemical status of biomarkers, including the phosphorylation of Rb and Ki67 in tumor tissue, was evaluated. Additional details for each experiment are given in the figure legends.

### Statistical analysis

All statistical analyses were performed using the JMP software package (version. 11.1.1). Continuous variables are expressed as the median and interquartile range or mean ± standard deviation. The Mann-Whitney U-test was used to compare continuous variables, and Fisher’s exact test was used to compare frequencies. A *p* value of < 0.05 was considered to be statistically significant.

## Results

### The effect of PD-0332991 on Rb related protein

**Fig 1:**
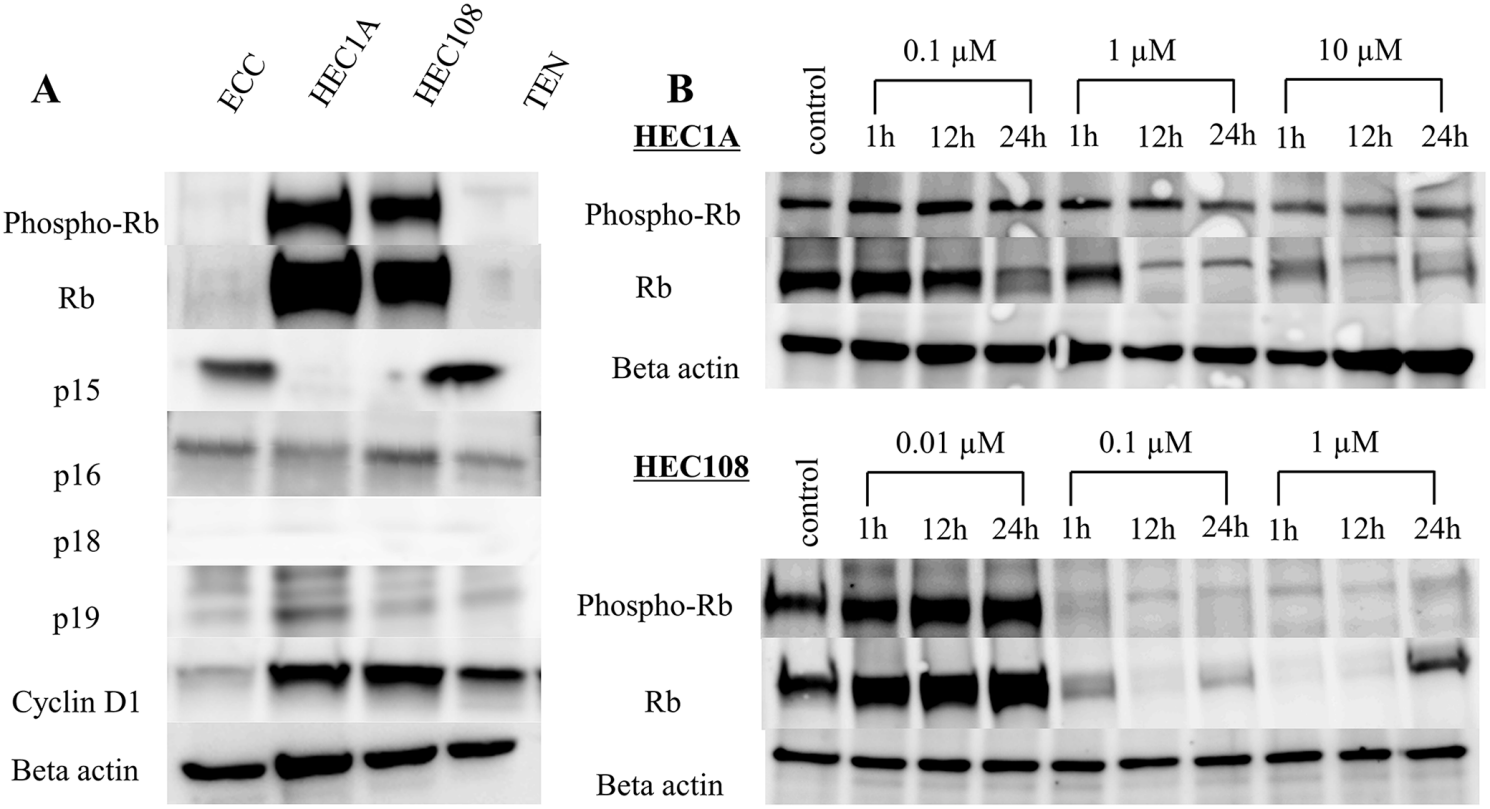
(A) A Western blot analysis shows the expression of Rb and phospho-Rb protein in HEC1A and HEC108 cells. Both cell lines demonstrated expression of cyclin D1. Strong expression of p15, p16, p18 and p19, which act as inhibitors of CDK 4/6, was not seen. (B) Effect of PD-0332991 on phosphorylation of Rb protein. HEC1A (top) and HEC108 (bottom) cells showed a time- and concentration-dependent inhibition of Rb phosphorylation following treatment with a PD-0332991 inhibitor. However, PD-0332991 inhibited total Rb expression to a greater extent than phospho-Rb.

Fig 1A shows the expression of Rb related protein in human endometrial cancer cell lines. HEC1A and HEC108 demonstrated expression of Rb and phospho-Rb protein. Both cell lines had apparent expression of cyclin D1 and weak to moderate expression of p16 and p19. Expression of p15 or p18 protein was not observed in these cell lines. Fig 1B shows Rb phosphorylation with increasing concentrations and exposure times of PD-0332991 in cell lines expressing Rb related protein. The inhibition of Rb phosphorylation was dependent on the concentration and exposure time of PD-0332991. However, PD-0332991 inhibited the total Rb expression to a greater extent than phospho-Rb.

### The effect of PD-0332991 on cell proliferation

**Fig 2:**
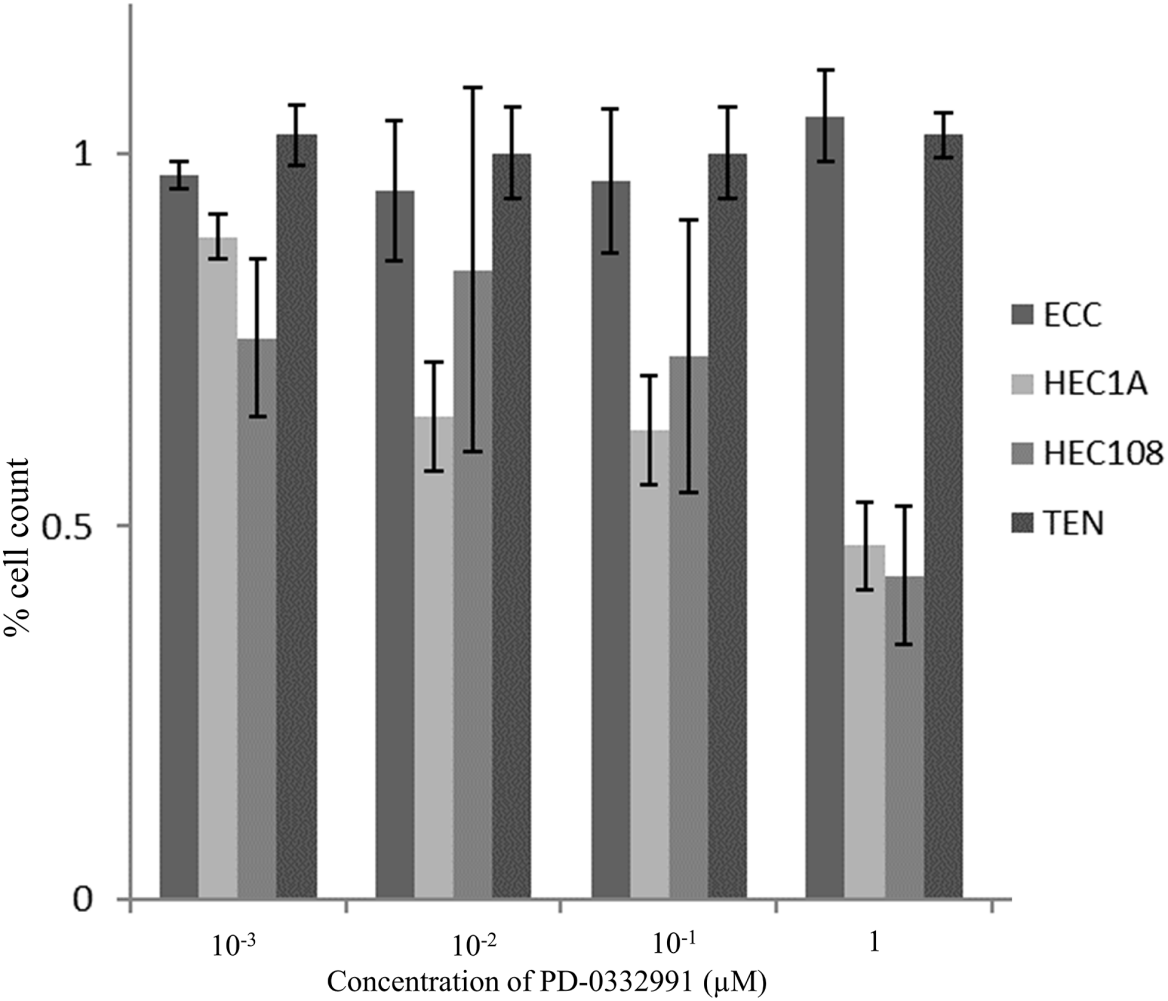
Proliferation ability of endometrial cancer cell lines following treatment with PD-0332991. Cells were plated onto 24-well tissue culture plates at a density of 1 × 10^4^ and grown without or with increasing concentrations of PD-0332991 for 72 hours. In HEC1A and HEC108 cells, PD-0332991 acted as a concentration-dependent inhibitor of cell proliferation with an IC50 of 0.65 and 0.58 μM, respectively. In ECC and TEN cells, however, PD-0332991 did not inhibit cell proliferation, even at concentrations up to 1 μM.

Fig 2 shows the proliferation ability of EC cell lines after treatment with increasing concentrations of PD-0332991 for 72 hours. In HEC1A and HEC108 cells, PD-0332991 acted as a concentration-dependent inhibitor of cell proliferation with an IC50 of 0.65 and 0.58 μM, respectively. In ECC and TEN cells, however, PD-0332991 did not inhibit cell proliferation, even at concentrations of up to 1 μM.

### The effect of PD-0332991 on cell cycle

**Fig 3:**
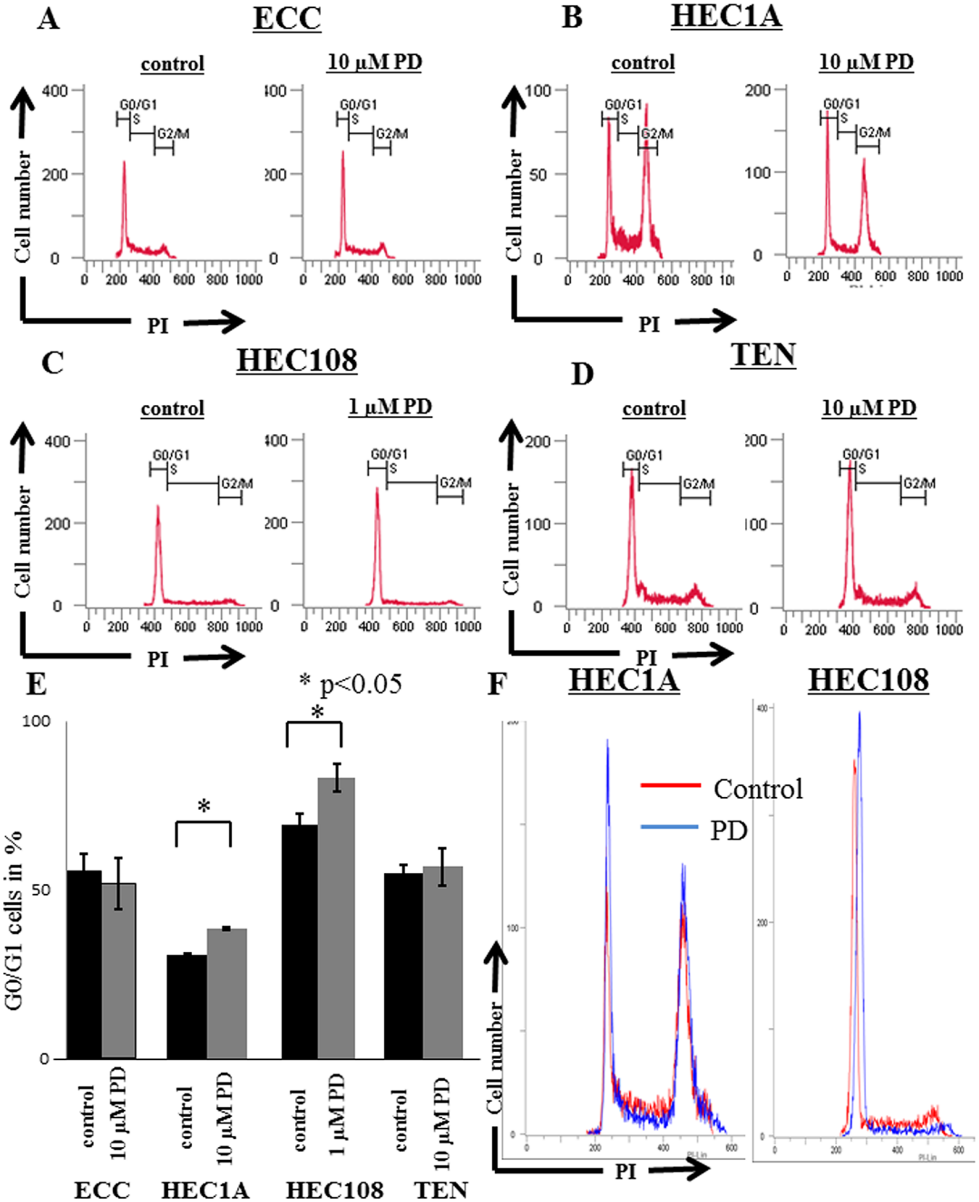
A cell cycle analysis of endometrial cancer cell lines treated with PD-0332991. The cells were harvested after being incubated for 72 hours in the absence or presence of 1 or 10 μM of PD-0332991, then cell proliferation was evaluated by measuring the distribution of cells in different phases of cell cycle by flow cytometry: **(A)** ECC; **(B)** HEC1A; **(C)** HEC108; and **(D)** TEN cells treated with control (left panel) or PD-0332991 (right panel). **(EF)** G0/G1 cell cycle arrest is apparent in HEC1A and HEC108 cells after treatment with PD-0332991.

**Fig 4:**
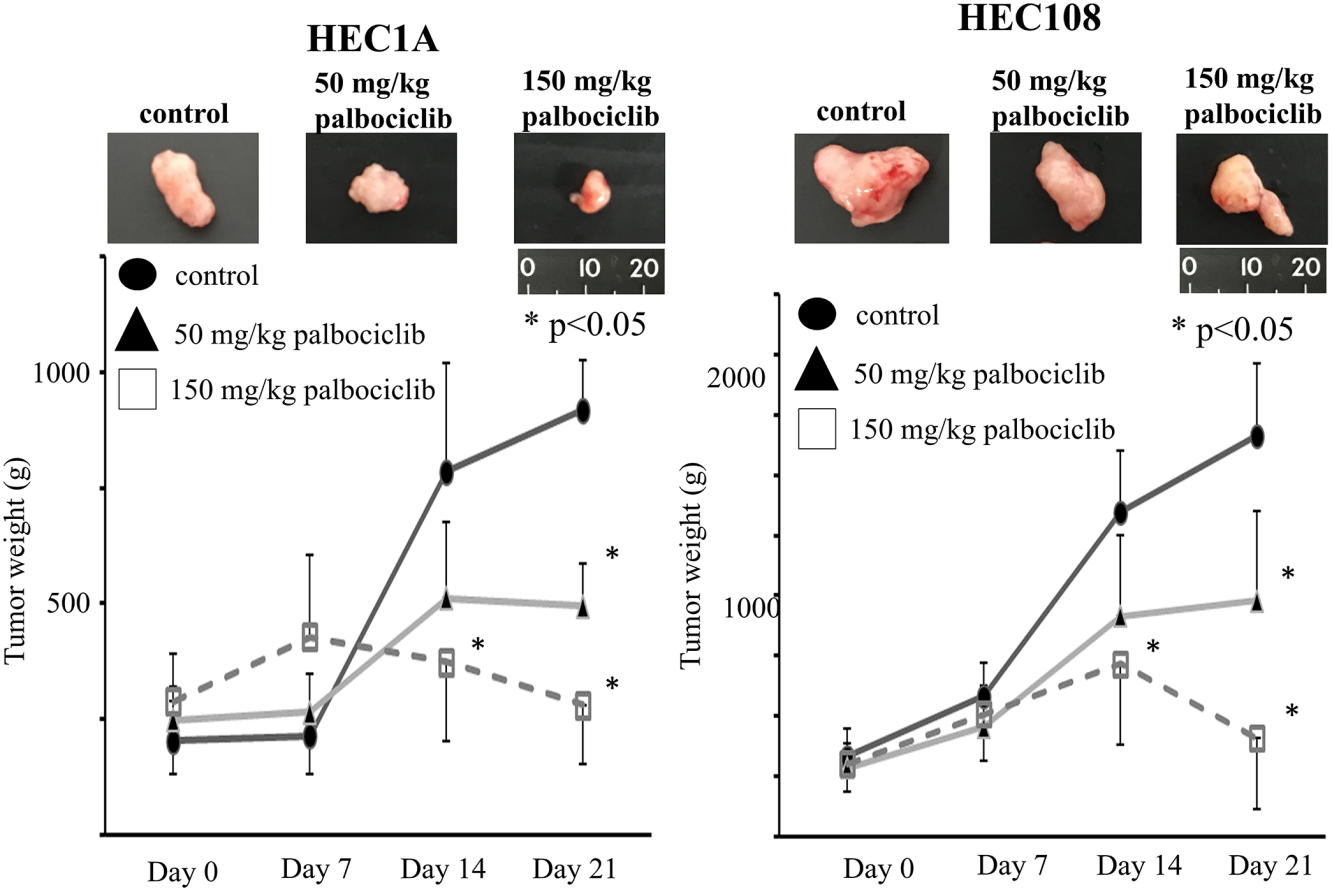
*In vivo* antitumor activity of PD-0332991 administered p.o. (A) Regression of HEC1A human endometrial cancer. Tumor fragments were implanted s.c. in nude mice and allowed to grow to at least 100 mg. PD-0332991 was given daily p.o. for 21 days by gavage at the indicated drug doses in lactate buffer (50 mmol/l) at pH 4.0. Untreated control (●), 50 mg/kg (▲) and 150 mg/kg (□). (B) Growth suppression of HEC108 human endometrial cancer. Tumor implantation and administration of PD-0332991 was done as in A. Untreated control (●), 50 mg/kg (▲) and 150 mg/kg (□). The photos show the tumors in each group after 21 days of treatment. In both cell lines, the tumor weights of the PD-0332991-treated group were significantly lower than those in the control group after 21 days of treatment (p<0.05). Furthermore, the tumor weights in the high-dose group (150 mg/kg) were significantly lower than those in the control group after 14 days of treatment (p<0.05)

Fig 3 shows the cell cycle analysis of endometrial cancer cells treated with PD-0332991. In ECC cells, the ratio of G0/G1 cells without or with 10 μM PD-0332991 treatment were 55.6% and 51.8% (p>0.05), respectively (Fig 3A and 3E). In HEC1A, these ratios were 30.9% and 38.9% (p<0.05), respectively (Fig 3B, 3E and 3F). However, 1 μM PD-0332991 did not increase the ratio of G0/G1 cells (data not shown). In HEC108, 1 μM PD-0332991 increased the ratio of G0/G1 cells (69.6% vs 83.4%, p<0.05) (Fig 4C, 4E and 4F). In TEN cells, 10 μM PD-0332991 did not increase the ratio of G0/G1 cells (55.2% vs 57.1%) (Fig 3D and 3E).

### The effect of Rb related protein status on tumor grade and invasiveness

Table 1 shows the characteristics of 337 Japanese patients with endometrial cancer. The mean (± SD) age of the patients was 58.0 ± 11.0 years of age. The mean body mass index was 23.8 ± 4.6 kg/m^2^. Two hundred and twenty-five (66.8%) patients were in FIGO stage I, 11 (3.3%) were in stage II, 65 (19.3%) were in stage III and 21 (6.2%) were in stage IV. Histologically, 17 (5.0%) patients had atypical endometrial hyperplasia, 226 (67.1%) patients had endometrioid carcinoma of grade 1 or 2, 45 (13.4%) had endometrioid carcinoma of grade 3, 24 (7.1%) had carcinosarcoma, 16 (4.7%) had serous carcinoma and 11 (3.3%) had clear cell carcinoma. Table 2 shows the association between the malignant potential of the tumor and immunochemical staining of Rb, phospho-Rb, p16 and Cyclin D1. Among these patients, 241 (71.5%) had low grade tumors including AEH and endometrioid carcinoma of grade 1 or 2, 96 (28.5%) had high grade tumors including endometrioid carcinoma of grade 3, carcinosarcoma, serous carcinoma and clear cell carcinoma. When AEH or < 50% myometrial invasion without metastasis were considered as no deep invasive cancer and tumors with metastasis or ≥ 50% myometrial invasion as deep invasive cancer, 190 (56.4%) patients had no deep invasive cancer and 147 (43.6%) had deep invasive cancer. The positivity rates of Rb, phospho-Rb, p16 and cyclin D1 were 67.7%, 32.6%, 84.0% and 52.5%, respectively. Two hundred and forty-four (72.4%) patients had tumors with positive Rb or phospho-Rb staining. In these patients, 17 (5.0%) had tumors with negative p16 and positive cyclin D1 staining, which is considered to be more sensitive to PD-0332991. Rb, phospho-Rb and p16 were not significantly associated with the tumor grade. However, cyclin D1-positive tumors had a higher rate of high grade tumors than cyclin D1-negative tumors (33.9% vs 22.5%, respectively, p = 0.02). Rb-positive tumors had a higher rate of invasive cancer than Rb-negative tumors (47.8% vs 34.9%, respectively, p = 0.02). In contrast, phospho-Rb-positive tumors had a lower rate of invasive cancer than phospho-Rb-negative tumors (28.2% vs 51.19%, respectively, p<0.01). p16 and cyclin D1 were not associated with tumor invasion (Table 2). The expression of Rb (S1 Fig), phospho-Rb (S2 Fig), p16 (S3 Fig) and cyclin D1 (S4 Fig) were not significantly associated with the prognosis, including the progression-free survival and overall survival.

**Table 1:** Characteristics of patients with endometrial cancer.

Number of patients	337
Age*	58.0 ± 11.0
BMI*	23.8 ± 4.6
FIGO stage	
I	225 (66.8)
II	11 (3.3)
III	65 (19.3)
IV	21 (6.2)
Histological type	
AEH	17 (5.0)
Endometrioid G1 or G2	224 (66.5)
Endometrioid G3	45 (13.4)
Carcinosarcoma	24 (7.1)
Serous	16 (4.7)
Clear cell	11 (3.3)

*According to an ANOVA (mean ± SD); BMI, body mass index; AEH, atypical endometrial hyperplasia.

**Table 2:** Immunohistochemical analysis of patients with endometrial cancer.

	Total	Rb		Phospho-Rb		P16		Cyclin D1	
		positive	negative	positive	negative	positive	negative	positive	negative
Number of patients	337	228 (67.7)	109 (32.3)	110 (32.6)	227 (67.4)	283 (84.0)	54 (16.0)	177 (52.5)	160 (47.5)
Grade									
Low	241 (71.5)	159 (69.7)	82 (75.2)	72 (65.5)	169 (74.5)	202 (71.4)	39 (72.2)	117 (66.1)*	124 (77.5)*
High	96 (28.5)	69 (30.3)	27 (24.8)	38 (34.6)	58 (25.6)	81 (28.6)	15 (27.8)	60 (33.9)*	36 (22.5)*
Invasion									
No deep invasion	190 (56.4)	119 (52.2)*	71 (65.1)*	79 (71.8)*	111 (48.9)*	154 (54.4)	36 (66.7)	100 (56.5)	90 (56.3)
Deep invasion	147 (43.6)	109 (47.8)*	38 (34.9)*	31 (28.2)*	116 (51.1)*	129 (45.6)	18 (33.3)	77 (43.5)	70 (43.8)

*Significant difference between the groups (p<0.05); Low grade, endometrioid carcinoma G1 or G2; High grade, endometrioid carcinoma G3, carcinosarcoma, serous carcinoma and clear cell carcinoma; No deep invasion, < 50% myometrial invasion without metastasis; Deep invasion, ≥50% myometrial invasion

### Anticancer activity of PD-0332991 in vivo

**Fig 5:**
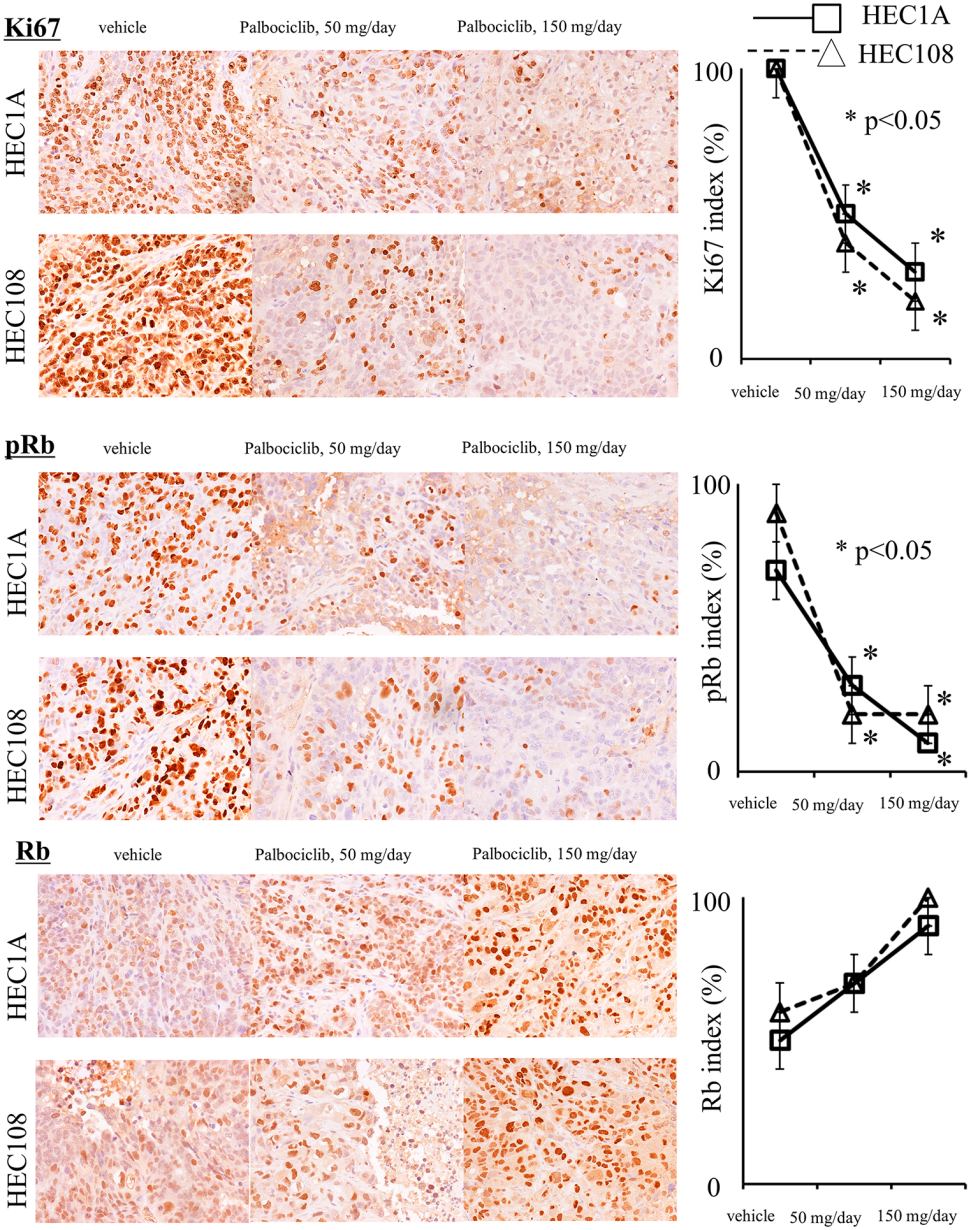
An immunohistochemical analysis of HEC1A (□) and HEC108 (Δ) human endometrial cancer tumors in model mice treated with PD-0332991. The Ki-67 index was lower in the PD-0332991-treated group than in other groups. Furthermore, the PD-0332991-treated group had higher Rb expression and lower phospho-Rb expression than the control group.

PD-0332991 exhibited antitumor efficacy against subcutaneous human endometrial cancer models. In mice bearing human endometrial cancer, daily p.o. dosing for 21 days with PD-0332991 (50 or 150 ng/kg) suppressed tumor growth (Fig 4). Fig 5 shows the results of the immunohistochemical analysis of the tumors in mice. The Ki67 index in the PD-0332991-treated group was significantly lower than that in the control group (p<0.05). Furthermore, the phospho-Rb expression of the PD-0332991-treated group was significantly lower than that in the control group (p<0.05). The PD-0332991 group showed a higher level of Rb expression; however, the difference was not statistically significant (p = 0.4). These findings suggest that PD-0332991 caused cell cycle arrest by inhibiting the phosphorylation of the Rb protein.

## Discussion

PD-0332991 has therapeutic potential in endometrial cancer cell lines expressing Rb protein. In our immunochemical analysis of 337 clinical specimens, the rate of tumors with positive Rb or phospho-Rb expression was 72.4%. In these patients, 17 (5.0%) patients had tumors with negative p16 and positive cyclin D1 expression. There were no significant relationships between the expressions of these proteins and the tumor grade.

PD-0332991 is a selective CDK 4/6 inhibitor that induces cell cycle arrest with reduced Rb phosphorylation [8]. The compounds CDK 4/6 and cyclin D1 release transcription factor E2F from Rb, which results in phosphorylation of Rb and subsequent cell proliferation [9, 10]. INK4 family members, including p15, p16, p18 and p19, act inhibitors of CDK 4/6 [11, 12]. In basic research, the efficacy of PD-0332991 had been reported in many kinds of cancer cell lines. In breast cancer, cell cycle arrest following PD-0332991 treatment is apparent in Rb-positive but not Rb-negative cell lines and xenografts [8, 13, 25]. Cell lines representing the luminal ER-positive subtype (including those that are HER2 amplified) were most sensitive to growth inhibition by PD-0332991. A gene expression analysis revealed that Rb and cyclin D1 were elevated and p16 was decreased in most sensitive cell lines [13]. In glioblastoma multiform, the response to PD0332991 failed to correlate with the level of CDK6 or 4 expression. Cell lines with Rb1 deletion were highly resistant, whereas cell lines with CDK amplification, p16 deletion or p16/p18 deletion were sensitive [14]. In prostate cancer, the efficacy of PD-0332991 was dependent on the Rb status, not on the androgen receptor. Investigation in this study demonstrated the ability of PD-0332991 to function in the presence of existing hormone-based regimens and cooperate with ionizing radiation to further suppress cellular growth [16]. In epithelial ovarian cancer, Rb proficient cell lines with low p16 expression were most responsive to CDK4/6 inhibition. Copy number variations of p16, Rb, cyclin E, and cyclin D1 were associated with the response to PD-0332991. Rb proficiency with low p16 expression was seen in 37% of ovarian cancer patients and independently associated with a poor progression-free survival [15].

In the present study, Western blotting confirmed that total Rb as well as pospho-Rb was decreased after treatment with PD-0332991 in endometrial cancer cell lines that expressed Rb. *In vivo*, immunohistochemical analyses revealed that phospho-Rb was decreased after treatment with PD-0332991; however, total Rb was not decreased. Several studies have shown that Rb phosphorylation is inhibited in a time- and concentration-dependent manner, while the expression of total Rb is decreased following treatment with the PD-0332991 *in vitro* [15, 26]. Similarly, *in vivo*, immunohistochemistry revealed that Rb phosphorylation was inhibited and Ki67 was suppressed [27]. To the best of our knowledge, no studies have shown an increase in Rb after PD-0332991 treatment *in vivo*. In our study, the level of Rb expression in the PD-0332991-treated group was higher than that in the control group *in vivo*; however, the difference did not reach statistical significance. We consider it difficult to find the point at which the level of Rb increased while the level of phospho-Rb simultaneously decreased *in vitro*. The concentration and the time of exposure to PD-0332991 deeply influence this phenomenon. We hypothesize that only non-Rb-expressing cells remain after PD-0332991 treatment. Thus, most Rb-expressing cells show cell cycle arrest, and it may be difficult to harvest such cells *in vitro*.

According to these findings, several clinical studies have been designed to include several factors such as the Rb status, hormone receptor status, p16 expression and cyclin D1 amplification. An initial phase I trial of PD-0332991 monotherapy was scheduled for patients with Rb-positive solid tumors and non-Hodgkin’s lymphoma [17, 18]. A subsequent phase II trial was conducted for Rb-positive breast cancer using a 125 mg dose with 3 weeks on, one week off [19]. In this study, positive ER was the only factor associated with the response. Other factors, including the expression of Rb, the Ki-67 index, the loss of p16 and cyclin D1 amplification, were not associated with the response. On the other hand, a randomized phase II trial of PD-0332991 as a first-line treatment (PALOMA1/TRIO1) was scheduled in postmenopausal females with ER+/HER2- advanced breast cancer [20]. Initially, this trial had two separate cohorts; in cohort 1, patients were enrolled according to their ER+/HER2- biomarker status alone, whereas in cohort 2 they were also required to have cancers with cyclin D1 amplification, p16 loss, or both. This study demonstrated that the addition of palbociclib to letrozole significantly improved the progression-free survival of ER+/HER2- breast cancer. However, the trial stopped further patient enrollment into cohort 2 because an interim analysis suggested that further patient selection according to cyclin D1 amplification and p16 loss was unlikely to further improve the patient outcome over the use of the ER and HER2 status alone. Furthermore, differences in the PFS between cohort 1 and cohort 2 suggested that cyclin D1 amplification and p16 loss may not be predictive for the response CDK 4/6 inhibition, but may be prognostic.

PALOMA2, a double-blind, phase III trial of palbociclib plus letrozole vs placebo plus letrozole as a first-line treatment of postmenopausal patients with ER+/HER2- advanced breast cancer was confirmatory and should be reported soon. PALOMA3, a double-blind phase III trial of palbociclib plus fulvestrant vs placebo plus fulvestrant in the second-line setting, was stopped early due to efficacy issues [21].

In germ cell tumors, phospho-Rb is commonly observed in more differentiated tumors such as teratomas. A phase II study of palbociclib was scheduled for patients with germ cell tumors including mature or immature teratomas, mature teratomas with malignant transformation and malignant germ cell tumors, such as embryonal carcinoma, yolk sac tumor, choriocarcinoma and seminoma. The authors concluded that treatment with palbociclib was associated with a favorable 24-week progression-free survival rate in patients with refractory germ cell tumors. A benefit was mainly observed in patients with unresectable teratoma and teratoma with malignant transformation [28].

## Conclusions

PD-0332991 was thus found to have a therapeutic potential in endometrial cancer cell lines expressing Rb protein. Our immunohistochemical analysis revealed that approximately 70% patients with endometrial cancer might have therapeutic indications for PD0332991. In contrast, the tumor grade had no impact on the indications for treatment.

## Supporting information

S1 Fig The prognosis of endometrial cancer patients with Rb expression. The expression of Rb were not significantly associated with the progression free survival and overall survival.
(TIF)

S2 Fig The prognosis of endometrial cancer patients with pRb expression. The patients with pRb-negative tumor had a poor progression free survival, however, the expression of pRb were not significantly associated with the overall survival.
(TIF)

S3 Fig The prognosis of endometrial cancer patients with p16 expression. The expression of p16 were not significantly associated with the progression free survival, however, the patients with p16-positive tumor had a poor overall survival.
(TIF)

S4 Fig The prognosis of endometrial cancer patients with CD1 expression. The expression of CD1 were not significantly associated with the progression free survival and overall survival.
(TIF)
